# VISIONET: intuitive visualisation of overlapping transcription factor networks, with applications in cardiogenic gene discovery

**DOI:** 10.1186/s12859-015-0578-0

**Published:** 2015-05-01

**Authors:** Hieu T Nim, Milena B Furtado, Mauro W Costa, Nadia A Rosenthal, Hiroaki Kitano, Sarah E Boyd

**Affiliations:** Systems Biology Institute (SBI) Australia, Monash University, Clayton, VIC 3800 Australia; Australian Regenerative Medicine Institute, Monash University, Clayton, VIC 3800 Australia; National Heart and Lung Institute, Imperial College London, London, W12 0NN UK; Sony Computer Science Laboratories, Inc., Higashigotanda, Shinagawa, Tokyo Japan; Okinawa Institute of Science and Technology, Onna, Onna-son, Kunigami, Okinawa Japan

**Keywords:** Visualisation, Human-readable, Gene expression, Transcription factor, Network overlap

## Abstract

**Background:**

Existing *de novo* software platforms have largely overlooked a valuable resource, the expertise of the intended biologist users. Typical data representations such as long gene lists, or highly dense and overlapping transcription factor networks often hinder biologists from relating these results to their expertise.

**Results:**

VISIONET, a streamlined visualisation tool built from experimental needs, enables biologists to transform large and dense overlapping transcription factor networks into sparse human-readable graphs via numerically filtering. The VISIONET interface allows users without a computing background to interactively explore and filter their data, and empowers them to apply their specialist knowledge on far more complex and substantial data sets than is currently possible. Applying VISIONET to the Tbx20-Gata4 transcription factor network led to the discovery and validation of *Aldh1a2*, an essential developmental gene associated with various important cardiac disorders, as a healthy adult cardiac fibroblast gene co-regulated by cardiogenic transcription factors Gata4 and Tbx20.

**Conclusions:**

We demonstrate with experimental validations the utility of VISIONET for expertise-driven gene discovery that opens new experimental directions that would not otherwise have been identified.

**Electronic supplementary material:**

The online version of this article (doi:10.1186/s12859-015-0578-0) contains supplementary material, which is available to authorized users.

## Background

A substantial body of computational research in biology is focused on building “*de novo* discovery platforms”, *i.e.* software that draws statistical predictions from high-throughput experiments [1]. In contrast, typical analyses by experimental biologists exploit specialist knowledge and an ability to accurately judge the feasibility of *in vivo* laboratory validations. The complementary power of computing and human expertise [2] has promoted a new class of expertise-driven semi-automated computational tools [3], such as the image analysis platform CL-Quant by Nikon that has recently gained commercial and clinical success [4,5].

Experimental biologists are usually intimately familiar with a finite set of genes featured in their biological system of interest. Key regulatory genes have generally already been investigated and confirmed *in vivo*, but the relationship between those genes and the transcription factors that regulate their expression is often unknown [6]. Transcription factors often act in concert, forming tightly controlled networks, and many gene targets are shared among different transcription factors [7,8]. Identification of overlapping regulation of genes within transcription factor networks carries significant potential for untangling the complex biological processes being studied.

Data visualisation is one of the most powerful approaches designed to bridge the computational/experimental divide and facilitate biological discovery, in particular visualisation of gene regulatory networks, where novel systems-level properties can be inferred from the network characteristics [1,9]. However two major hurdles still persist for biologists; different types of -omics assays cannot be integrated, greatly limiting the utility for biologists [10], and the sheer scale of these the networks exceeds capacity for human interpretation [11].

We have encountered exactly this scenario in the field of cardiac research. The mammalian heart is a complex structure with highly specialised cells working under a tightly regulated environment [7,8]. To understand cardiac function and disease, the cardiovascular research community still largely uses conventional approaches (*e.g*. transgenic mice) and thus focuses on a small group of highly cardiac-specific genes [12]. Their data sets are generally very focussed, in-house, and specific to particular experimental conditions. Working hypotheses are generally based on the existing body of literature, on substantial in-house expertise, and on an experimental approach that is optimised for the research being undertaken within that group or laboratory. The missing link between their experimental research and computational approaches is a tool that facilitates mapping of gene expression data onto transcription factor networks.

We have therefore developed VISIONET, a tool to integrate transcription factor (TF) networks obtained from ChIP-seq studies with gene expression levels from microarray data. The purpose of this tool is to allow biologist users to apply domain expertise to reason about and explore the experimental data that they have generated. In particular, VISIONET is designed to reveal co-regulated genes that have strong expression signatures. Unlike other typical data-intensive tools for analysing ChIP-Seq or microarray data, VISIONET is specifically designed for biologists, with a web-based interactive graphical interface tailored to provide human-readable information, which filters the dense network according to gene expression levels and displays reduced quantities of information to facilitate direct interpretation by the expert users. We have also implemented customised layout algorithms that are specific for TF networks, and in particular overlapping TF networks, which are optimized for human readability. VISIONET is intended as a complementary tool to the existing large-scale discovery platforms such as Cytoscape and CellDesigner.

We illustrate the purpose and utility of our tool with a case study in which we have applied VISIONET to microarray results obtained in our recent investigation of TFs that regulate cardiac fibroblasts identity [13]. In the developing mouse heart, Tbx20 directly interacts with Gata4 to co-regulate the heart development program [14] and Tbx20-Gata4 co-regulated genes are increasingly important topics for systematic investigation [15]. We have revisited this dataset using VISIONET, to show that integrated visualisation of the Gata4 and Tbx20 TF networks allows rapid discovery of common co-regulated genes in the adult mouse cardiac fibroblasts. This approach has led to identification of *Aldh1a2*, a gene that already has a recognised role in cardiogenesis [16], and now from this study also appears to be highly up-regulated in adult cardiac fibroblasts.

## Implementation

**Figure 1:**
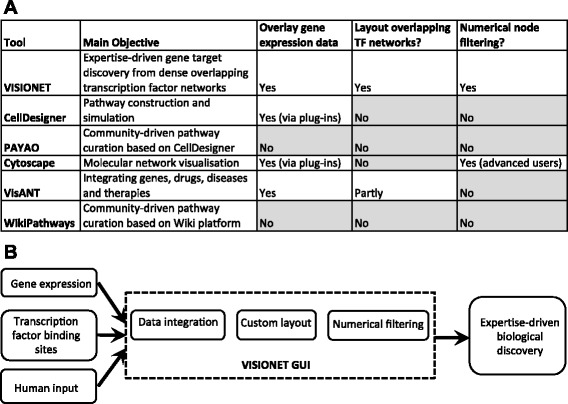
VISIONET implementation uniqueness. **(A)** Comparison of VISIONET features with popular biological network visualisation analysis tools. VISIONET implements five features to facilitate expertise-driven visualisation and analysis of overlapping transcription factor networks. Using empirical evaluation based on our case study, the availability of these features was assessed in the most popular existing tools: CellDesigner v4.4 [17], PAYAO [18], Cytoscape v2.8 [19], VisANT 4.0 [20], and WikiPathways [21]. **(B)** Schematic flowchart illustrating the architecture of VISIONET, designed to facilitate expertise-driven biological discovery. Gene expression and TF binding site data are supplied to VISIONET, and biologist interacts with VISIONET to determine which network components to display. Internally, VISIONET performs the computationally intensive task of data integration, graph layout and network filtering.

The design of the VISIONET system is based on satisfying the requirements of expertise-driven gene discovery, which requires all of the following features: overlaying gene expression data on top of transcription factor networks, layout methods tailored to visualising overlapping transcription factor networks, and numerically filtering for human readability. In our experience working in cardiac biology, these features (Figure 1A) are not all currently available in existing interactive visualisation platforms CellDesigner v4.4 [17], PAYAO [18], Cytoscape v2.8 [19], VisANT 4.0 [20], and WikiPathways [21]. In the specific case of TF network topology, currently it is not a trivial task to generate a readable network layout and apply numerical filtering using existing visualisation platforms. All existing platforms do not have specific layout method for overlapping TF network topology. Numerical filtering, if available (such as in Cytoscape) requires a substantial degree of user sophistication, much greater than the average level of a general biologist user.

The VISIONET web service was developed in the Microsoft ASP.NET environment using the open source NodeXL application programming interface [22]. The VISIONET pipeline (Figure 1B) has a back-end that handles the data integration and graph rendering from the transcriptomic datasets, and a front-end for biologist users that allows them to interactively control the display of the TF network. The node properties can be any numerical values that the biologist users are measuring (fold-change, *p*-values, RNA-Seq’s reads per kilobase, *etc*.). Thus VISIONET enables users to address the overall biological question (gene discovery) and specific biological questions (genes having certain fold-changes, *p*-values, *etc*.). The biologist users can interact with the web-based graphical interface from most common browsers in at least two ways: by defining what properties to be associated with each node (fold-change, *p*-value of a *t*-test), and by specifying the cut-off for numerical filtering.. The network graphics are rendered in the *GIF* format, which has excellent compression ratio for images with few distinct colours.

Two input files are required: (1) the *< gene 1, gene 2 >* tuples that describe the TF network, and (2) the *< gene, value >* tuples for the microarray intensity. Inputs (1) and (2) are provided in tab- or comma-separated format, and (1) also accepts *GraphML* format [23]. Alternatively, the user can supply the raw ChIP-Seq peak list for (1) in the standard *COD* format, and the raw microarray files for (2) in the standard *SOFT* format.

The filtering feature of VISIONET enables the user to control the visualisation of the network, so that only a small (relevant) sub-network of interest is displayed, while the remainder of the network is blurred or omitted. This feature is highly useful for biologists, as human inspection is feasible when only a small number of genes are visible at a time. Filtering is typically performed calculating the log fold change (Log FC) value of each gene based on the supplied transcriptomes to VISIONET. Optionally, other filtering criteria are possible by supplying VISIONET with a list of < *gene, value* > tuples that represents any numerical property of the genes. Thus there are numerous use cases for VISIONET filtering function, such as “filter out all genes with Log FC value between −4 and 4”, or “show only genes with *p*-value < 0.001 based on an unpaired *t*-test”.

VISIONET has a customised layout algorithm that takes advantage of the topology of TF networks, where each edge connects a high-degreed node (TF) and numerous low-degreed nodes (target genes). In brief, the layout algorithm spaces the TFs equidistant from each other in a circle, and layout the target genes randomly in fixed-location boxes (Algorithm 1).

The performance of the VISIONET web service depends on the number of nodes (i.e. genes) and number of edges (i.e. gene interactions) in the network. A typical network size for most ChIP-Seq datasets is ~7400 nodes and ~7400 edges (note that most nodes have *degree* = 1), where each TF has several thousands of binding sites. A network of this size can be rendered in by VISIONET in less than one minute on our server.

A companion desktop version of VISIONET is available as a Microsoft Excel add-in. Since Excel is proprietary software, this desktop version is provided solely as an additional convenience to users, further to the web service. The GraphML format enables users to work interchangeably between the web-based and desktop versions of VISIONET, and is convertible to the standard SBML format via the GraphMLReader plug-in for Cytoscape [24].

## Results

**Figure 2:**
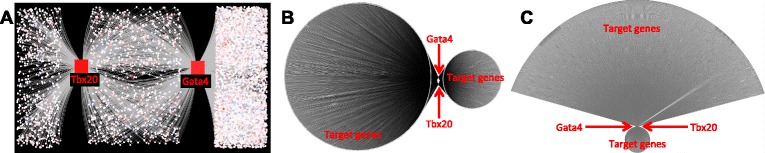
Comparison of VISIONET with the current state-of-art visualisation platforms, using the Gata4-Tbx20 case study. **(A)** Network using the customised VISIONET layout. **(B)** Network rending by Cytoscape using the “grouped by degree” layout (the most readable layout among other Cytoscape layouts in our empirical testing). **(C)** Network rending by CellDesigner using the “Circular” layout (the most readable layout among other CellDesigner layouts in our empirical testing).

Previously, VISIONET has been applied to the Gata4-Gata6 transcription factor network to discover *Hand2*, a developmental gene co-regulated by Gata4 and Gata6, being highly up-regulated in the adult cardiac fibroblasts [3]. Here, we applied VISIONET to construct cardiac fibroblast TF networks for Gata4 and Tbx20 (Figure 2A), two important cardiogenic TFs in heart development [25,26]. Not only have Gata4 and Tbx20 been shown to co-regulate important cardiac structure and functions during development [14,27] and in adult mice [28], we have made the recent surprising finding that both TFs are among the most highly up-regulated TFs in adult cardiac fibroblasts [13].

TFs have numerous gene targets, and frequently co-regulate gene expression. Identifying overlapping gene regulation, especially using visual approaches, is particularly interesting to biologists. In the case of cardiac function, the overlapping regions between the Gata4 and Tbx20 sub-networks are important for uncovering their largely unknown roles in the adult cardiac fibroblasts, because these two transcription factors are already known to co-regulate critical functions during heart development [14,15,27]. Existing platforms do not currently provide good solutions to this task. We illustrate this with the popular Cytoscape and CellDesigner platforms, which currently provide the most comprehensive libraries of layout methods. While VISIONET effectively visualises the overlapping target genes using its customised layout algorithm (Figure 2A), even the best empirical visual representations from Cytoscape (Figure 2B) and CellDesigner (Figure 2C) do not effectively display overlapping TF networks.

**Figure 3:**
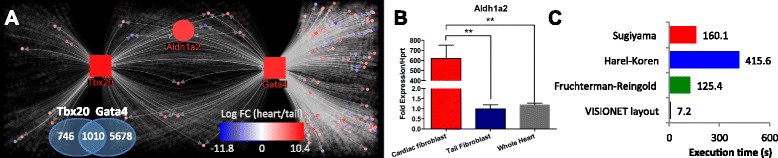
Experimental validation of the utility of the expertise-driven gene discovery approach. **(A)** Overlapping TF networks of Gata4 and Tbx20 in cardiac fibroblasts generated by VISIONET, with a filter applied to blur out all gene with Log FC(heart/tail) value between −4 and 4. Node colours were determined according to the heart/tail fibroblast fold-change obtained from microarray data [13]. Gata4, Tbx20, and *Aldh1a2* were labelled and enlarged, and other node labels were omitted, for improved visualisation. Squares indicate TFs and circles indicate target genes. The full list of differentially-expressed genes is shown in Additional file 1: S1. Log FC(heart/tail): Log_2_ of the fold change between heart and tail fibroblast expression. The Venn diagram shows the number of targets (based on ChIP-Seq peaks) of the Tbx20 and Gata4. **(B)** qPCR validation that the *Aldh1a2* gene is uniquely up-regulated in cardiac fibroblasts. Means and standard deviations (*n* = 3) are shown, and (**) indicates *p*-value < 0.01 (unpaired *t*-test). **(C)** Execution time (seconds) precise to 1 decimal place of the VISIONET web service for the Tbx20-Gata4 network using different layout algorithms. All algorithms were implemented in the same programming language and tested on the same computational hardware for comparison consistency.

We used VISIONET to generate the overlapping TF networks of Tbx20 and Gata4, from the ChIP-Seq data (materials and methods described in Additional file 1: Text S1), and then overlaid our own microarray data of heart and tail fibroblasts [13] to highlight the expression levels; the resulting network can be seen in Figure 3A. To understand the heart-specific properties of cardiac fibroblasts, we must use fibroblasts from another organ as a reference. Together with cardiac fibroblasts, tail fibroblasts were previously reported to be reprogrammable into heart muscle cells [29]. This makes heart and tail fibroblasts important subjects for therapeutic applications in heart regeneration, and therefore highly interesting to compare.

A common feature of ChIP-Seq experiments is the thousands of ChIP-Seq peaks that are generated for each TF, leading to very large networks. The Gata4-Tbx20 co-regulation network contains 7434 nodes (Figure 3A). Without filtering, this network would be too dense and complex for human analysis (Figure 2A). We therefore used the VISIONET Log Fold Change (Log FC) filtering option to highlight genes that have at least 16-fold differences between heart and tail fibroblast expression levels. Genes that are up-regulated in the cardiac fibroblast relative to the tail fibroblast, i.e. Log FC > 4, were coloured solid red; genes that are relatively up-regulated in the tail fibroblast (Log FC < −4) were coloured solid blue; and all other genes are coloured grey and blurred out. This filtering and highlighting renders a human-readable graph, to which biologists can apply expert analysis (Figure 3A, solid nodes).

The filtered network revealed that out of the ~7400 genes in the entire network, only 13 genes (Additional file 1: Table S2) have 16-fold differences in expression between heart and tail fibroblasts, and are co-regulated by Tbx20 and Gata4. From these 13 genes, we then concentrated on the cardiogenic gene *Aldh1a2* (also known as *Raldh2*). The *Aldh1a2* gene stands out to cardiac experts because it uniquely displays three features: it is co-regulated by both Tbx20 and Gata4, it is more than 16-fold up-regulated in the cardiac fibroblasts compared to tail fibroblasts, and its mutation is known to be highly associated with diverse congenital heart disease phenotypes, including Tetralogy of Fallot and Pentalogy of Cantrell [30,31]. It is also significant in the context of our research program, because *Alhd1a2* is an essential gene for heart development [32,33], and its presence in the cardiac fibroblasts strongly reinforces our recent findings of a cardiogenic gene expression signature in the normal adult cardiac fibroblasts [13]. We therefore used qPCR to confirm that *Aldh1a2* is indeed strongly up-regulated in heart fibroblasts, but not in tail fibroblasts or the whole heart (Figure 3B, and Additional file 1: Text S1).

In addition to our customised TF layout algorithm, we also provide implementations of popular open-source layout algorithms from the NodeXL API, including Fruchterman-Reingold [34], Harel-Koren [35], and Sugiyama [36]. For the Gata4-Tbx20 case study, the customised VISIONET layout method was substantially faster than other layout methods (Figure 3C), while still providing a human-readable display (Figure 3A).

## Discussion

Transcription factors often act in concert to co-regulate genes, and therefore the overlap of multiple TF networks carries important biological implications [7,8,37]. VISIONET is designed to be a simple but powerful visualisation tool for biologists to study these overlapping networks. Our tool brings together a number of key features that enable expertise-driven discovery, including allowing biologist users to interact directly with the TF network and determine the components of the network that are highly relevant to the biological question. The overlapping TF networks can be supplied, or derived, by using straightforward algorithms, and filtering based on microarray data can subsequently reduce the complexity of the network.

VISIONET was developed in-house and tailored to our biological question of cardiac fibroblasts characterisation, but is nevertheless broadly suitable for expertise-driven biological discovery. In a VISIONET graph, the network topology represents the transcription factor networks constructed from ChIP-Seq datasets, and the node colours represent the transcriptomic profile obtained from microarray experiments. The transcriptomic profile can then be used as a filter to limit the number of nodes visible to the biologist users, allowing the identification of important genes based on human expertise. Although microarray values may not be easily inferred solely from the node colours, users can interactively set the threshold for VISIONET to only display nodes above (or below) a certain value. By limiting the number of visible genes in the network, researchers can then apply their expertise to identify strongly relevant or unexpected transcripts, as we demonstrated with *Aldh1a2* (Figure 3A-B) and *Hand2* [3].

Being custom-made, VISIONET has features not yet addressed by other popular general-purpose visualization platforms, including CellDesigner and Cytoscape. There exists a myriad of visualisation platforms that provides a partial list of features provided by VISIONET (Figure 1A), but none have provided the streamlined user experience tailored to the specific task of expertise-driven discovery, as provided by VISIONET. This has been reinforced by our experience of uncovering the common target of Tbx20 and Gata4 in the adult cardiac fibroblasts (Figure 3A-B), where the discovery of *Aldh1a2* would not have been achieved using other visualisation platforms (Figure 2).

The majority of Tbx20 targets (58%) are also Gata4 targets (Figure 2B), consistent with other studies showing that Gata4 and Tbx20 interact to regulate heart structure and function in development [14,27]. The identification of the *Aldh1a2* gene as a target of Tbx20 based on ChIP-Seq data agrees with our previous findings that cardiac fibroblasts in Tbx20 conditional knockout mice displayed reduced *Aldh1a2* activity [13]. Furthermore, *Aldh1a2* is an established direct downstream target of Gata4 [38,39], as confirmed by our Tbx20-Gata4 case study using VISIONET.

Biological network visualisation has been an active area of research, and VISIONET is also designed to continuously improve its limitations and to adapt to the changing technological landscape. In our Gata4-Tbx20 case study, we have applied VISIONET for two overlapping TF networks (Figure 3A-B) due to the nature of the experiment, but the customised layout algorithm can also accommodate any larger number (>2) of TF overlapping networks. We currently implemented VISIONET using the NodeXL API for the node filtering support and web accessibility. Since the popular platforms CellDesinger [40] and Cytoscape are widely used, future work will also develop VISIONET as a plug-in for CellDesigner and/or Cytoscape. Also, as VISIONET depends on input ChIP-Seq and microarray data, it also inherits the technological pitfalls of these transcriptomic technologies. The peak list of ChIP-Seq contains uncertainties in the TF target gene information, and our microarray values may not indicate accurately the *in vivo* gene expression level [41].

## Conclusions

VISIONET is an in-house and streamlined tool for the specific tasks of expertise-driven gene discovery, with an implementation of features that are not all concurrently available in the popular comprehensive analysis platforms. In concert with other computational tools under development by the systems biology community, VISIONET bridges the gap between complex dataset and biologist users for a better understanding of biological systems.

## Availability and requirements

**Project name:** VISIONET

**Project home page:**http://VISIONET.erc.monash.edu.au

**Source code, desktop companion software and tutorials:**http://VISIONET.erc.monash.edu.au

**Operating system(s):** Platform independent

**Programming language:** C#, ASP.NET

**Other requirements (desktop application only):** Microsoft Excel 2007 or later.

**License:** GNU GPL

**Any restrictions to use by non-academics:** none

## Additional file

Additional file 1:
**Supplementary text and table.**

## Abbreviations

VISIONET: Visualisation of overlapping transcription factor network
TF: Transcription factor
ChIP-Seq: Chromatin immunoprecipitation sequencing
qPCR: quantitative real-time polymerase chain reaction
