# Supplementary material for: VISIONET: intuitive visualisation of overlapping transcription factor networks, with applications in cardiogenic gene discovery
Source: BMC Bioinformatics. 2015 May 1;16(1):141. doi: 10.1186/s12859-015-0578-0 (PMC4426166; doi:10.1186/s12859-015-0578-0)
Supplement: Additional file 1: — Supplementary text and table. [file 12859_2015_578_MOESM1_ESM.pdf]

VISIONET: Intuitive visualisation of overlapping transcription factor networks, with applications in cardiogenic gene discovery

Hieu T. Nim[1,2*], Milena B. Furtado[2], Mauro W. Costa[2], Nadia A. Rosenthal[1,2,3], Hiroaki Kitano[1,2,4,5] and Sarah E. Boyd[1,2*]

[1]Systems Biology Institute (SBI) Australia, Monash University, VIC 3800, Australia
[2]Australian Regenerative Medicine Institute, Monash University, VIC 3800, Australia
[3]National Heart and Lung Institute, Imperial College London, W12 0NN UK
[4]Sony Computer Science Laboratories, Inc., Higashigotanda, Shinagawa, Tokyo, Japan
[5]Okinawa Institute of Science and Technology, Onna, Onna-son, Kunigami, Okinawa, Japan
[*]Corresponding to: hieu.nim@monash.edu; sarah.boyd@sbiaustralia.org

SUPPLEMENTARY METHODS AND MATERIALS

Text S1. Materials and Methods

Animal ethics statement

All mice were kept in a full BL6-J background, housed at Monash Animal Services and experiments conformed with requirements under the ethics application MARP-2011-175 (Monash University).

Sample preparation, microarray and qPCR

The mouse cardiac and tail fibroblast cell preparation, microarray and RT-qPCR assays were performed as previously described [1]. The mouse Aldh1a2 primers for RT-qPCR experiments were: for 5'-TGCAGGCTGGGCTGATAAAA; rev 5'- GTGAACATCAGCAGGGGGAA. Transcription factor networks for Gata4 and Tbx20 were constructed from ChIP-Seq datasets available from NCBI GEO with accession numbers GSM558904 and GSM734426, respectively. The microarray data for the cardiac fibroblasts and tail fibroblasts are available from NCBI GEO with accession number GSE50531 [1]. The raw ChIP-Seq datasets were downloaded from NCBI in *.BED format, and then annotated using the CisGenome package [2] to produce an output file in *.COD format. Raw microarray datasets were downloaded from NCBI in *.TXT format, and then converted to *.SOFT format by selecting the appropriate columns using Microsoft Excel.

Table S2. List of 13 genes (>16-fold different between heart and tail fibroblasts) that are co-regulated by Tbx20 and Gata4.

Gene Names	LogFC(Heart/Tail)
Aldh1a2	7.904636667
Heyl	6.3092139
Tgm2	5.544541333
Pde1a	5.356449333
Nr1h4	5.2692862
Tnnt2	4.803914333
Kif26b	4.619946067
Ch25h	4.576112867
Chst11	4.551474767
Ptprb	4.461602167
Mtus2	4.126453833
Actc1	-4.990979567
Pcp4l1	-6.8415073

REFERENCES

1. Furtado MB, Costa MW, Pranoto EA, Salimova E, Pinto AR, Lam NT, Park A, Snider P, Chandran A, Harvey RP *et al*: **Cardiogenic genes expressed in cardiac fibroblasts contribute to heart development and repair**. *Circulation research* 2014, **114**(9):1422-1434.
2. Ji H, Jiang H, Ma W, Johnson DS, Myers RM, Wong WH: **An integrated software system for analyzing ChIP-chip and ChIP-seq data**. *Nature Biotechnology* 2008, **26**(11):1293-1300.
